# Supplementary material for: Non-linear dimensionality reduction on extracellular waveforms reveals cell type diversity in premotor cortex
Source: eLife. 2021 Aug 6;10:e67490. doi: 10.7554/eLife.67490 (PMC8452311; doi:10.7554/eLife.67490)
Supplement: Transparent reporting form [file elife-67490-transrepform.docx]

***eLife’s* transparent reporting form**

We encourage authors to provide detailed information *within their submission* to facilitate the interpretation and replication of experiments. Authors can upload supporting documentation to indicate the use of appropriate reporting guidelines for health-related research (see [EQUATOR Network](http://www.equator-network.org/%20)), life science research (see the [BioSharing Information Resource](https://biosharing.org/)), or the [ARRIVE guidelines](http://www.plosbiology.org/article/info:doi/10.1371/journal.pbio.1000412) for reporting work involving animal research. Where applicable, authors should refer to any relevant reporting standards documents in this form.

If you have any questions, please consult our Journal Policies and/or contact us: [editorial@elifesciences.org](mailto:editorial@elifesciences.org).

**Sample-size estimation**

- You should state whether an appropriate sample size was computed when the study was being designed
- You should state the statistical method of sample size computation and any required assumptions
- If no explicit power analysis was used, you should describe how you decided what sample (replicate) size (number) to use

Please outline where this information can be found within the submission (e.g., sections or figure legends), or explain why this information doesn’t apply to your submission:

We used two monkey subjects for the study as is standard. We also collected electrophysiological data from 75 sessions in Monkey T and 66 sessions in Monkey O and from > 100000 trials. For the questions considered in the study, the experiments are vastly overpowered. We analyzed the results of clustering 625 waveforms. In Supp. Fig. 2B we perform an explicit analysis of how our results depend on dataset size. W e showed that we needed only ~400 waveforms to observe the results identified in the manuscript.

**Replicates**

- You should report how often each experiment was performed
- You should include a definition of biological versus technical replication
- The data obtained should be provided and sufficient information should be provided to indicate the number of independent biological and/or technical replicates
- If you encountered any outliers, you should describe how these were handled
- Criteria for exclusion/inclusion of data should be clearly stated
- High-throughput sequence data should be uploaded before submission, with a private link for reviewers provided (these are available from both GEO and ArrayExpress)

Please outline where this information can be found within the submission (e.g., sections or figure legends), or explain why this information doesn’t apply to your submission:

Trial numbers for animals, number of sessions, and total number of waveforms analyzed are reported in the legend of Fig. 1. We did not remove any outliers from the data. We excluded positive peak first waveforms because of their unknown origin and only included waveforms that are considered to be single neurons (based on well-accepted inter-spike interval criteria). This is described in section "Recordings and Single Neuron Identification" and in the methods in "Alignment and normalization of waveforms". BIC and other model selection criteria were used for identifying number of GMM clusters.

**Statistical reporting**

- Statistical analysis methods should be described and justified
- Raw data should be presented in figures whenever informative to do so (typically when N per group is less than 10)
- For each experiment, you should identify the statistical tests used, exact values of N, definitions of center, methods of multiple test correction, and dispersion and precision measures (e.g., mean, median, SD, SEM, confidence intervals; and, for the major substantive results, a measure of effect size (e.g., Pearson's r, Cohen's d)
- Report exact p-values wherever possible alongside the summary statistics and 95% confidence intervals. These should be reported for all key questions and not only when the p-value is less than 0.05.

Please outline where this information can be found within the submission (e.g., sections or figure legends), or explain why this information doesn’t apply to your submission:

All statistical tests are described in the Statistics section of the Methods. Numerical data is described as mean +- standard error (SE), mean +- standard deviation (SD), or median +- standard error of the median (SEM). Effect sizes are also listed as appropriate: Cohen's f2 for one-way ANOVAs, and adjusted R2 for regression, and variance explained in a linear model. Whenever multiple testing was incurred, a Benjamini-Hochberg correction was used.

These statistical tests can be found in sections "WaveMAP clusters have distinct physiological properties", "WaveMAP clusters have distinct decision-related dynamics", "WaveMAP clusters contain distinct Laminar Distributions", and “Heterogeneity in decision-related activity emerges from both cell type and layer”. In addition, supplementary Figs. 2-6 perform important controls to control for effects due to random seeds in our algorithms. All hyperparameters are documented in Table S1. Note the code provided below also provides full access to all data used for statistical tests.

(For large datasets, or papers with a very large number of statistical tests, you may upload a single table file with tests, Ns, etc., with reference to sections in the manuscript.)

**Group allocation**

- Indicate how samples were allocated into experimental groups (in the case of clinical studies, please specify allocation to treatment method); if randomization was used, please also state if restricted randomization was applied
- Indicate if masking was used during group allocation, data collection and/or data analysis

Please outline where this information can be found within the submission (e.g., sections or figure legends), or explain why this information doesn’t apply to your submission:

N/A

**Additional data files (“source data”)**

- We encourage you to upload relevant additional data files, such as numerical data that are represented as a graph in a figure, or as a summary table
- Where provided, these should be in the most useful format, and they can be uploaded as “Source data” files linked to a main figure or table
- Include model definition files including the full list of parameters used
- Include code used for data analysis (e.g., R, MatLab)
- Avoid stating that data files are “available upon request”

Please indicate the figures or tables for which source data files have been provided:

Code for figures and analyses in Python are available as a Jupyter notebook (.ipynb) and are fully replicable. Other figures generated in MATLAB are deposited to a Github repository and are replicable with as a Live Script (.mlx). Associated processed data files are also available within the repository. Non-default parameters and random forest classifier hyperparameters are listed in Supplementary Table S1.
